# Discovery of *Typhonium mukdahanense* (Araceae), a New Species from Northeastern Thailand

**DOI:** 10.3390/life16081376

**Published:** 2026-08-20

**Authors:** Wilawan Promprom, Phukphon Munglue, Chuthep Phannasri, Wannachai Chatan

**Affiliations:** 1 Department of Biology, Faculty of Science, Mahasarakham University, Maha Sarakham 44150, Thailand; wilawan.pp@msu.ac.th (W.P.);; 2 Plant and Innovation Research Unit, Mahasarakham University, Maha Sarakham 44150, Thailand; phukphon.m@ubru.ac.th; 3 Program of Biology, Faculty of Science, Ubon Ratchathani Rajabhat University, Ubon Ratchathani 34000, Thailand

**Keywords:** aroid, Indochina, nomenclature, plant diversity

## Abstract

*Typhonium* is one of the most species-rich genera of Araceae in tropical Asia, with north-eastern Thailand representing an important but still incompletely documented area of diversity. During botanical surveys in northeastern Thailand, an unusual population of *Typhonium* was discovered in mixed deciduous forest and dry evergreen forest at 300–400 m elevation. Detailed morphological study of living plants and herbarium specimens, together with comparisons with relevant protologues, regional floras, and recently described species, confirmed that this population represents a species new to science. It is here described as *Typhonium mukdahanense* Chatan & Promprom, sp. nov. The new species is morphologically most similar to *T. fornicatum*, but differs by its taller habit, larger hastate to trisect leaf blades, more numerous primary lateral veins, larger and nearly symmetrical spathe tube, triangular reflexed spathe limb, dark red stigma with a cream-colored depressed center, sterile interstice bearing 10–14 staminodes arranged in two whorls, slightly curved and not spreading–declinate appendix, and pyriform mottled berries. It also differs from *T. acetosella* by its broader leaf blades, reflexed triangular spathe limb, dark red stigma, and pyriform fruits. The new species is currently known only from the type locality and is therefore provisionally assessed as Critically Endangered (CR) according to the IUCN Red List Categories and Criteria. A detailed description, diagnosis, information on distribution, habitat, phenology, conservation status, and an identification key to morphologically similar species are provided.

## 1. Introduction

*Typhonium* Schott represents one of the most species-rich genera within the tribe Areae and is primarily distributed in tropical Asia, with a center of diversity in Indochina and adjacent regions. Recent taxonomic treatments recognize approximately 70–100 species worldwide [1]. Nevertheless, the true diversity of the genus is probably underestimated, owing to the presence of numerous narrowly endemic, localized, and poorly documented taxa.

The genus *Typhonium* is not only taxonomically diverse but also ethnobotanically and pharmacologically interesting. Several species have been reported in traditional medicine systems of Asia for the treatment of inflammation, respiratory disorders, gastrointestinal complaints, skin diseases, swellings, and venomous bites [2,3]. Previous studies have shown that some species, such as *T. trilobatum* and *T. blumei*, possess bioactivities including analgesic, anti-inflammatory, antidiarrheal, and anti-allergic effects [4,5]. These activities may be associated with diverse secondary metabolites, including flavonoids, phytosterols, fatty acids, and related bioactive compounds [5].

Thailand is an important center of diversity for *Typhonium*. The Flora of Thailand treatment recognized more than thirty species and highlighted the country as a hotspot for diversification within the genus [6]. During the past decade, several new species have been described from Thailand, such as *T. vinicolor* P.Saensouk, K.Z.Hein & Saensouk [7] and *T. fornicatum* P.Saensouk, K.Z.Hein & Saensouk [8], and other narrowly distributed endemic taxa, indicating that the diversity of the genus remains incompletely documented. These discoveries suggest that additional undescribed species are likely to occur in underexplored habitats, particularly within northeastern Thailand.

Ongoing discoveries of new Araceae species and new distributional records from Phu Pha Yol National Park and surrounding areas highlight the high, but still incompletely documented, diversity of the family in this region of Thailand. Recently documented taxa, including *Colocasia sookchaloemiae* Chatan & Promprom, and *Pycnospatha phanomdongrakensis* Chatan & Promprom [9,10], provide further evidence that the aroid flora of Thailand remains incompletely known. This is especially relevant for taxonomically complex groups in which species delimitation relies on careful examination of subtle vegetative, floral, and reproductive characters.

During field surveys conducted in Phu Pha Yol National Park, Mukdahan Province, northeastern Thailand, an unusual population of *Typhonium* was discovered growing in mixed deciduous forest and dry evergreen forest. The plants exhibit a distinctive combination of characters. Detailed comparisons with herbarium specimens, protologues, taxonomic revisions, and recently described species from Thailand and neighbouring countries revealed that the collected material does not correspond to any previously known taxon. Morphologically, the new species most closely resembles *Typhonium fornicatum* P.Saensouk, K.Z.Hein & Saensouk [8], a species recently described in Bueng Kan Province in northeastern Thailand. However, the Mukdahan plants differ markedly in vegetative, floral, and fruit characteristics, particularly in the plant height, larger leaf blades, greater number of primary lateral veins, larger and nearly symmetrical spathe tube, triangular reflexed spathe limb, dark red stigma, numerous staminodes arranged in two whorls, not spreading-declinate appendix, and pyriform berries. These differences support recognition of the Mukdahan population as a distinct species.

The objective of the present study was to describe and illustrate a new species of *Typhonium* from northeastern Thailand and to provide information on its morphology, distribution, habitat, phenology, conservation status, and relationships with morphologically similar species.

## 2. Materials and Methods

Field studies were conducted in Phu Pha Yol National Park, Mukdahan Province, northeastern Thailand, and adjacent areas during 2024–2025. Living plants were observed and documented in their natural habitats, and selected specimens were temporarily maintained in a garden for observation during flowering and fruiting. Representative specimens were collected and prepared following standard botanical procedures, and type specimens were deposited in the BCU herbarium in Bangkok, Thailand (the collection number: W. Chatan 4513; the herbarium numbers: 1. Holotype A 18449; 2. Isotype A 18450). Herbarium acronyms follow [11].

In the laboratory, morphological observations and measurements were made from both living material and dried herbarium specimens. Vegetative and reproductive structures were measured using a ruler or digital calipers, whereas smaller floral features were examined under a stereomicroscope when more precise assessment was necessary. Terminology used in the taxonomic description follows Henk Beentje [12] and the treatment of Araceae in the Flora of Thailand [6]. Species delimitation was based on comparative evaluation of diagnostically informative morphological characters within *Typhonium*, particularly plant habit, leaf shape and venation, spathe morphology, the dimensions and arrangement of the floral zones of the spadix, the number and distribution of staminodes, stigma morphology, appendix characteristics, and fruit morphology.

Specimens of *Typhonium* from Thailand and neighbouring regions were examined either directly in herbaria or through high-resolution digital images available via JSTOR Global Plants and the online databases of the respective institutions [13]. Herbarium collections consulted included A, B, BCU, BK, BKF, BM, BRI, C, CNS, DNA, E, G, K, KATH, L, M, MEL, NSW, P, PERTH, SING, and UTB. Regional floristic accounts, taxonomic treatments, and other relevant literature were also consulted [6,8,14,15,16,17].

Morphological comparisons with the similar species were based on protologues, type specimens, or available images of the similar species *T. fornicatum* (Surapon Ara002, holotype, KKU) and *T. acetosella* Gagnep. (Kerr 21525, lectotype, K; isolectotype, BK) [8,17]. Habitat, elevation, phenology, and geographic coordinates were recorded during fieldwork to characterize the species distribution. Preliminary conservation status was assessed following the IUCN Red List Categories and Criteria [18].

## 3. Results

### 3.1. Taxonomic Treatment

**Figure 1:**
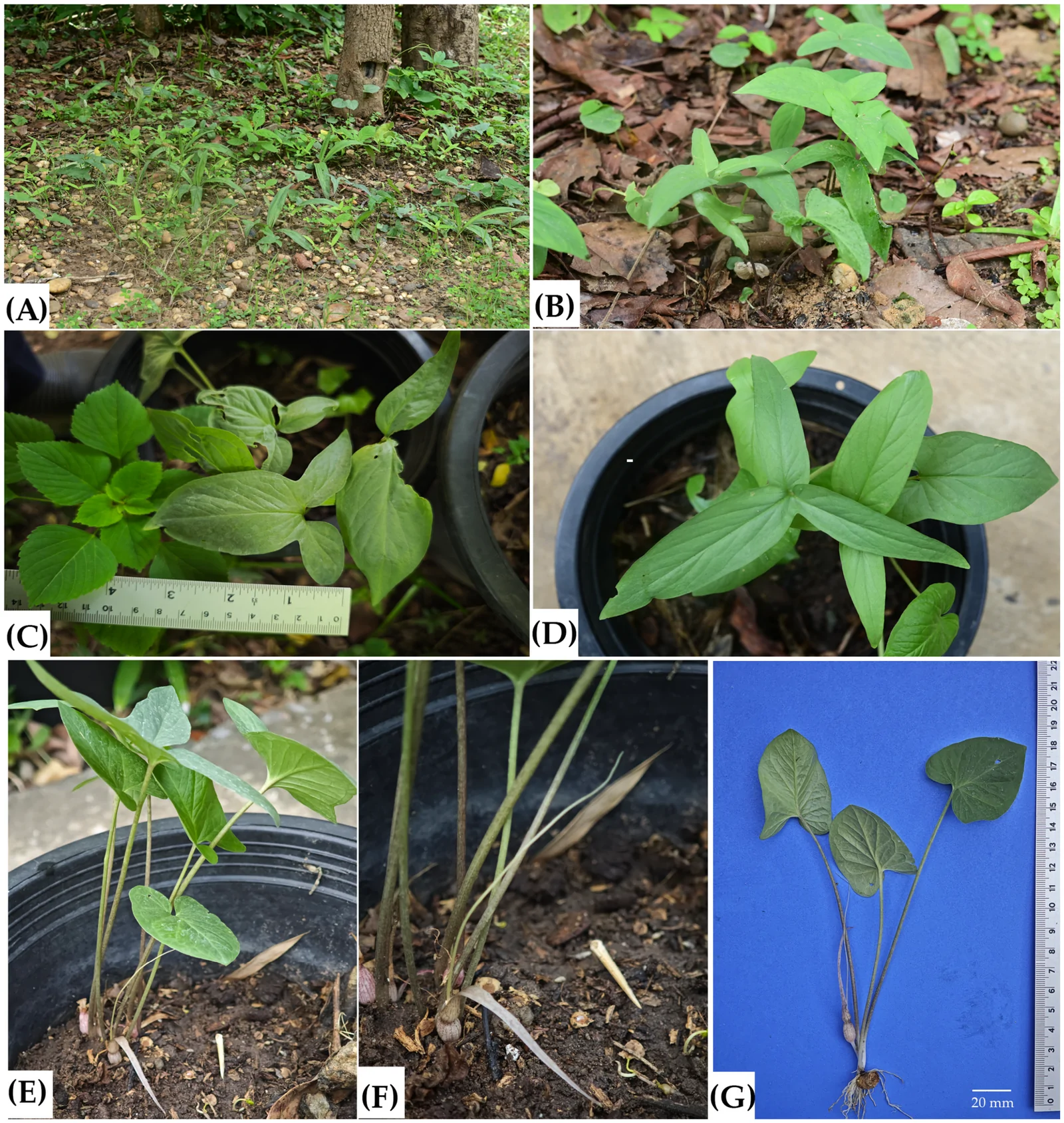
*Typhonium mukdahanense*. (**A**,**B**) Habitat and habit under natural field conditions; (**C**–**F**) Habit of cultivated plants; (**G**) Whole plant.

**Figure 2:**
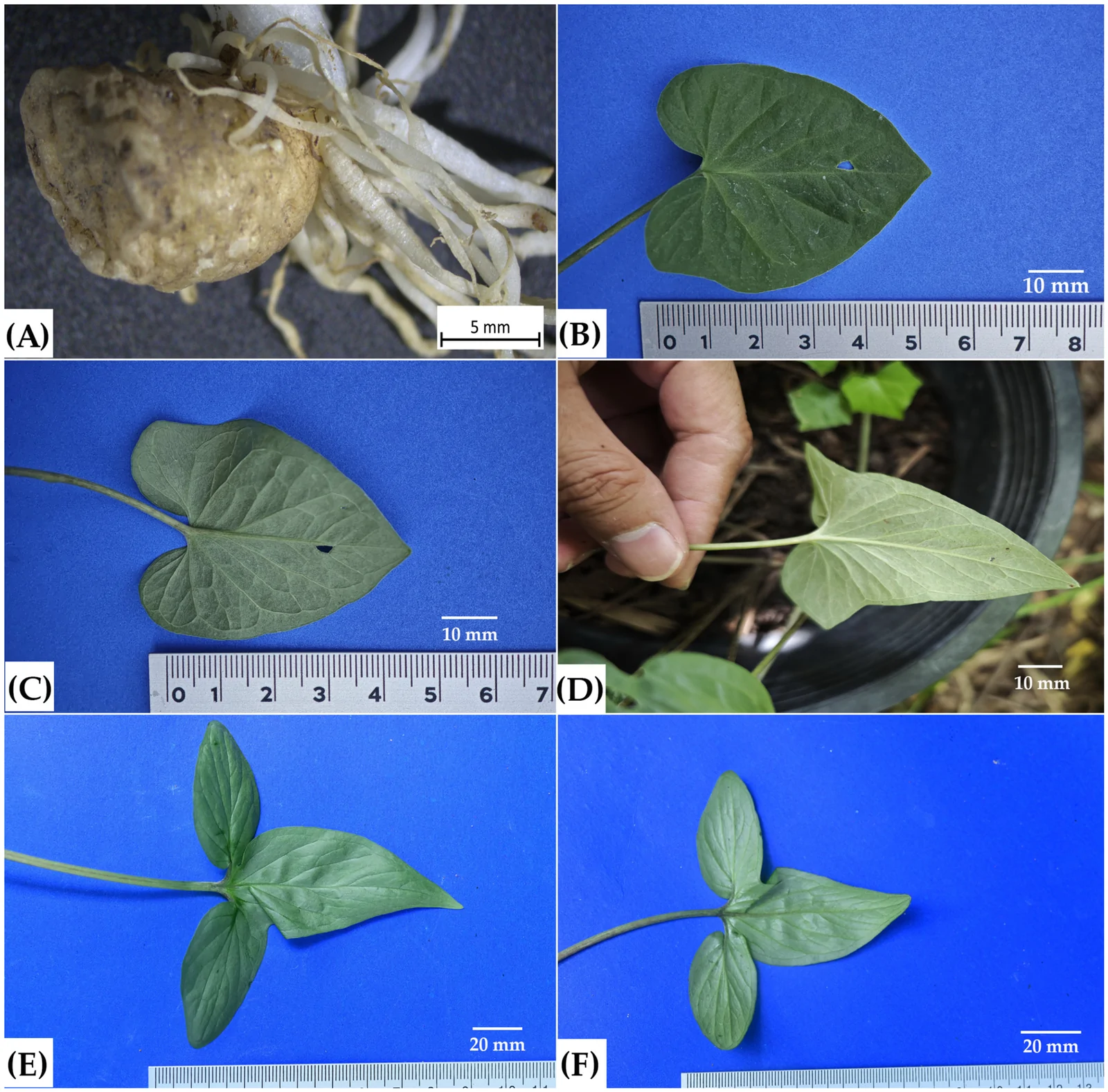
*Typhonium mukdahanense*. (**A**) Tuber and roots; (**B**,**E**) Leaf blades (adaxial surfaces); (**C**,**D**,**F**) Leaf blades (abaxial surfaces).

**Figure 3:**
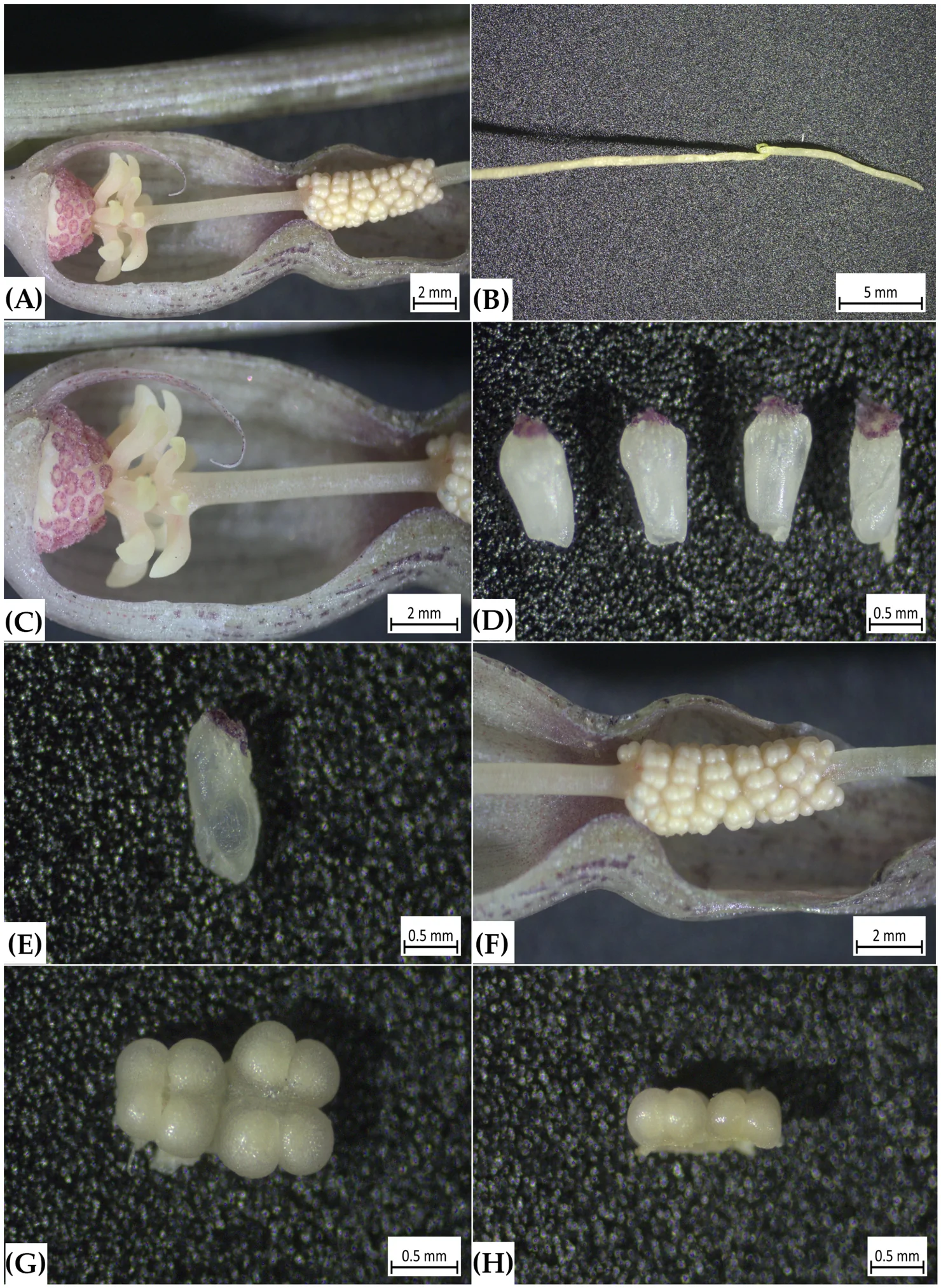
*Typhonium mukdahanense*. (**A**) Basal portion of the spadix showing the pistillate zone, staminodes, and sterile interstice; (**B**) Appendix; (**C**) Enlarged view of the pistillate zone, staminodes, and sterile interstice; (**D**) Pistils; (**E**) Enlarged view of a pistil; (**F**) Staminate zone; (**G**,**H**) Stamens in top and lateral views, respectively.

**Figure 4:**
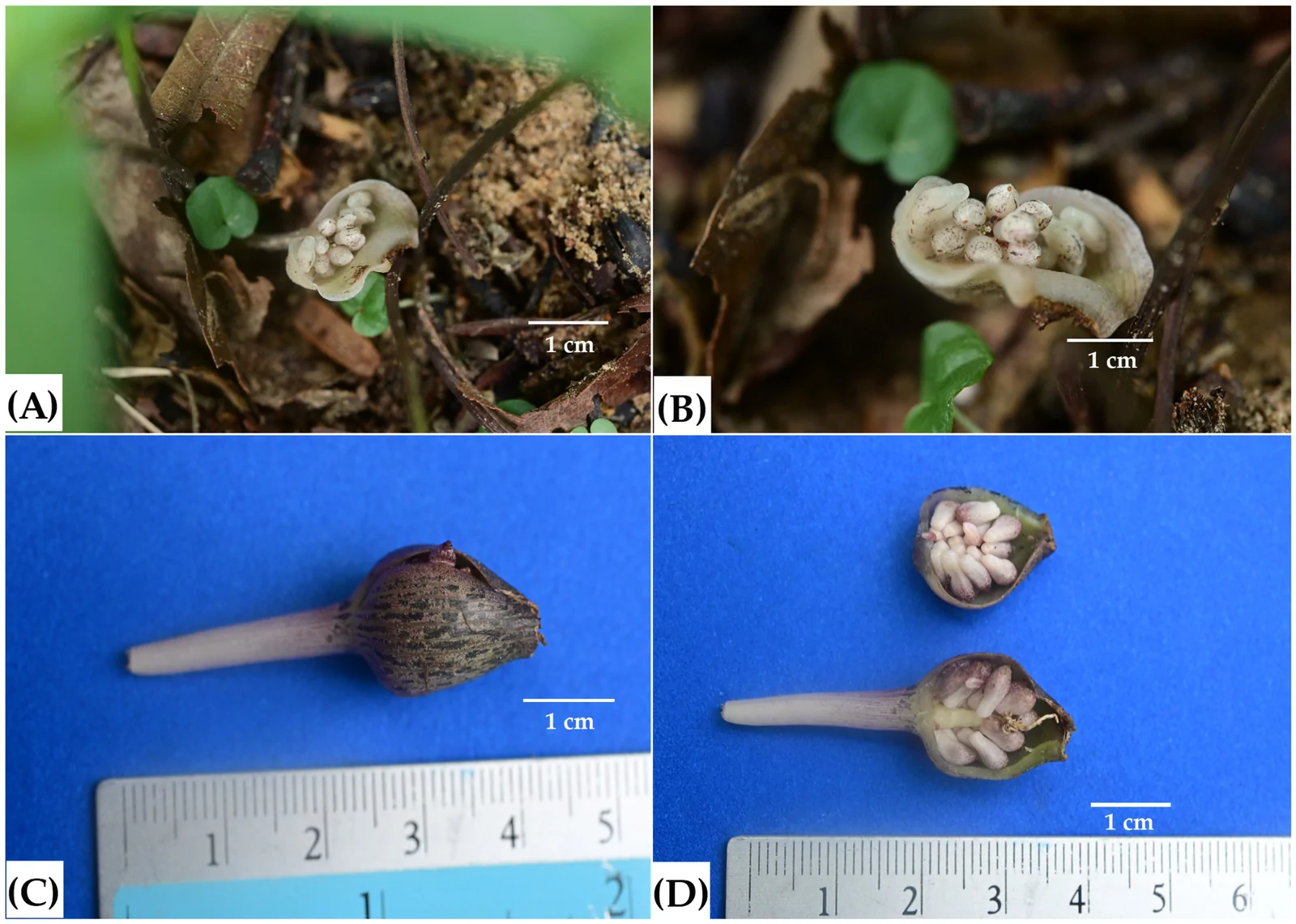
*Typhonium mukdahanense*. (**A**,**B**) Naturally damaged infructescence in the field; (**C**,**D**) Infructescence before and after longitudinal sectioning.

*Typhonium mukdahanense* Chatan & Promprom, sp. nov. (Figure 1, Figure 2, Figure 3 and Figure 4).

### 3.2. Type

Thailand. Northeastern: Mukdahan Province, 300–400 m elev., 16°53′35.3″ N 104°14′48.4″ E, 22 April 2024, W. Chatan 4513.

### 3.3. Diagnosis

*Typhonium mukdahanense* Chatan & Promprom, sp. nov., is most similar to *Typhonium fornicatum* in its clustered leaves and small inflorescences, but differs by its taller habit (up to 18 cm vs. up to 10 cm tall); larger hastate to trisect leaf blades (8–12 × 6.5–9.5 cm vs. 5.0–6.5 × 2.6–2.8 cm); more numerous primary lateral veins (5–8 vs. 3–4 per side of the midrib); larger and nearly symmetrical spathe tube (12–15 mm long vs. 8–9 mm long); triangular reflexed spathe limb with the adaxial surface facing upwards (vs. lanceolate and fornicate); longer spadix (9.5–10 cm vs. ca. 7 cm long); stigma dark red with a cream-colored depressed center (vs. white); sterile interstice bearing 10–14 staminodes arranged in two whorls (vs. only three staminodes arranged in single whorl); appendix slightly curved and not spreading-declinate (vs. spreading-declinate); and pyriform berries irregularly mottled or speckled with reddish- to purplish-brown markings (vs. clavate berries with darker brown mottling). A detailed morphological comparison between the new species and its most similar species is provided in Table 1.

### 3.4. Description

Seasonally dormant herbs, up to 18 cm high. Tuber hypogeal, subglobose or depressed globose, 1–2 cm across, pale brown externally and white internally. Roots thread-like, 0.5–1 mm across, white. Leaves 2–4 per plant; petioles slender, terete to slightly canaliculate, glabrous, 7–16 cm long, 1–2 mm in diameter; basal portion shortly sheathing, whitish green to pale pinkish purple, usually subterranean; lower half greenish brown to brownish purple with dark longitudinal streaks; upper half green to pale green, weakly streaked or nearly uniform. Leaf blade 8–12 × 6.5–9.5 cm, hastate or trisect or rarely ovate; thinly chartaceous, dull green adaxially, paler dull green abaxially, glabrous on both surfaces, with margins entire to trisect; anterior lobe apex of ovate leaf acute or acuminate; posterior lobes spreading outwards, apex obtuse; base cordate or slightly cordate; midrib adaxially impressed, abaxially raised, 1.5–2 mm broad at the base, gradually narrowing toward the blade apex; primary lateral veins 5–8 on each side, impressed adaxially and raised abaxially, anastomosing 1–5 mm from the margin to form an intramarginal collective vein; one marginal vein present; interprimary veins less prominent than the primary veins; higher-order venation reticulate. Inflorescence solitary, with a subtending cataphyll or cataphyll absent; cataphyll 2.5–3 cm long, linear-lanceolate, membranous and hyaline, initially white, becoming brown upon withering; peduncle 2–2.2 cm long, 1.5–1.8 mm across, mostly subterranean, white or pinkish, terete and glabrous; spathe clearly differentiated by a constriction into the spathe tube and spathe limb; spathe tube 12–15 mm in length, 7.6–7.9 mm in diameter, convolute, symmetrical or slightly asymmetrical ovoid, pinkish orange externally, tinged and streaked purple; internally whitish to pale cream, faintly purplish. Spathe limb 12–15 mm long, 7.6–7.9 mm in diameter at base, linear triangular, externally pinkish orange, tinged and streaked purple, internally whitish to pale cream, faintly purplish; base of the spathe limb shortly convolute; upper part erect, then reflexed, with the adaxial surface facing upward and the abaxial surface facing downward; margins entire with apex narrowly acute. Spadix sessile, 9.5–10 cm long, markedly exceeding the spathe in length; Pistillate zone 2.4–2.8 mm in length, 3.8–4.1 mm across at the base, shortly conical, comprising four rows of densely crowded pistils; ovary 0.95–1.1 mm high, 0.6–0.7 mm in diameter, clavate-oblong, white, unilocular, containing a single ovule attached to a basal placenta; stigma sessile, 0.5–0.55 mm across, discoid, slightly depressed at the center, dark red with a cream-colored center, papillate; sterile interstice 10–11.5 mm in length (including a 2.5–3 mm long staminode-bearing zone in the lower part), ca. 0.8–0.9 mm across, upper part naked, terete, glabrous and glossy white, lower part bearing two whorls of 10–14 staminodes; staminodes 1.8–2.0 mm in length, 0.6–0.8 mm across at the widest point, clavate–falcate with a slightly blunt apex and directed downwards, free, glabrous, cream or ivory; staminate zone 5–6 mm in length, 2–2.1 mm across, cylindrical to subcylindrical; stamens congested in clusters of two, each cluster 0.6–1 mm in diameter, cream to dull yellow-brown; anthers exceeding the connective. Appendix sessile, filiform, 8–9 cm in length, 0.8–1 mm across, very slender, elongated, slightly curved, tapering toward the apex, pale cream to very pale creamy brown, glabrous. Infructescence ovoid, 1.5–1.8 cm in length and 1.2–1.5 cm across at the widest point, pale purple to purple, densely streaked longitudinally with blackish-brown lines or spots; apex dark reddish-brown to purplish-brown; spathe tube persistent; peduncle 2.0–2.8 cm long, whitish to pale greenish brown. Berries pyriform 0.4–0.5 cm in length, 2–3 mm across at the widest point, apex obtuse to rounded, lower portion mostly cream or whitish, upper portion pinkish to pale purplish-brown, irregularly mottled or speckled with reddish-brown to purplish-brown markings.

### 3.5. Etymology

The specific epithet refers to its type locality, Mukdahan Province, in northeastern Thailand.

### 3.6. Vernacular Name

Buk Bai Mon.

### 3.7. Phenology

Flowering observed in April and July; fruiting observed in July and August.

### 3.8. Distribution and Habitat

**Figure 5:**
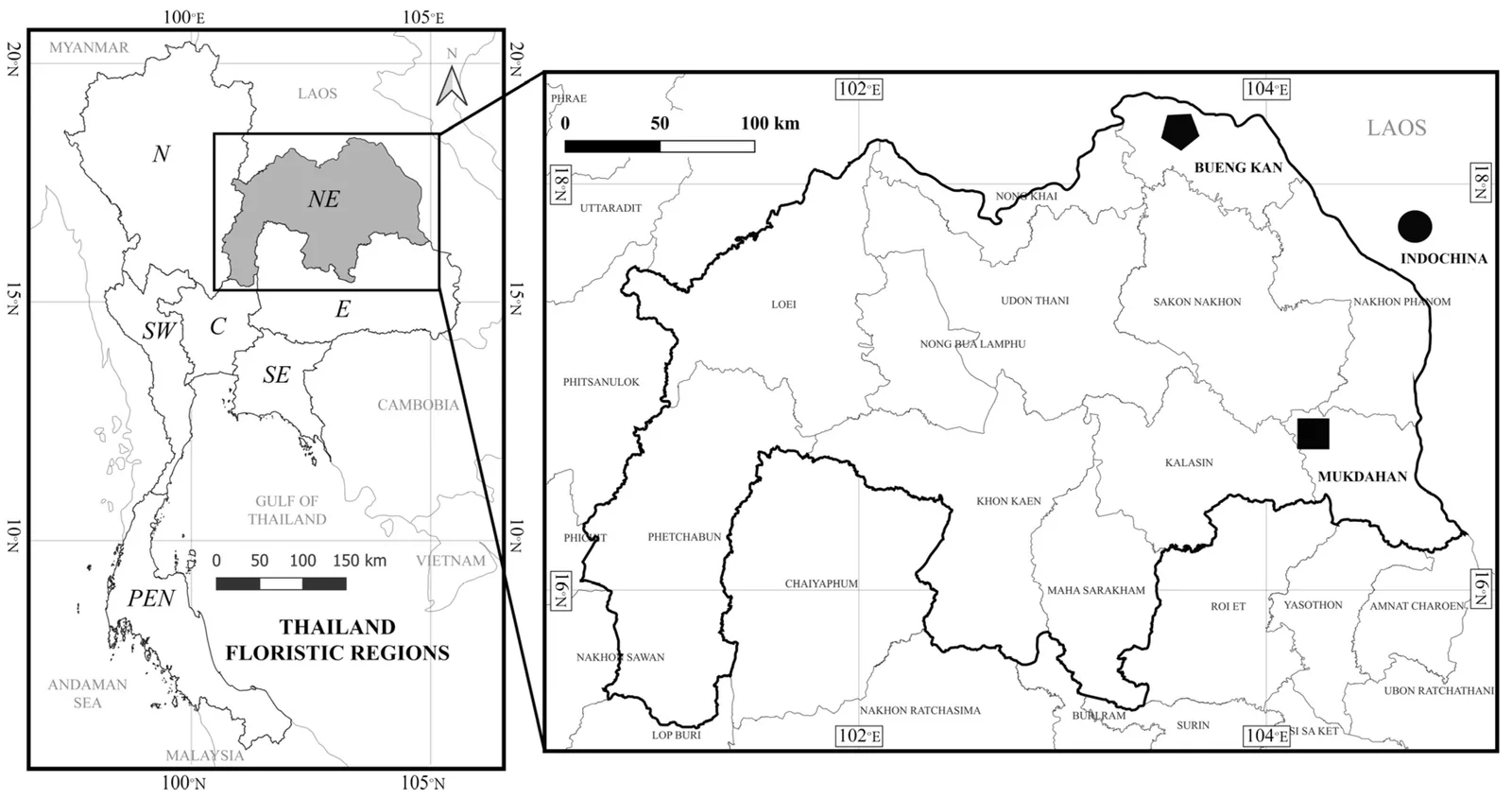
Distribution map of *Typhonium mukdahanense* in Mukdahan Province (black square) and *T. fornicatum* in Bueng Kan Province (black pentagon), in northeastern Thailand; *T. acetosella* in Indochina (black circle).

The new species is currently known only from its type locality in Mukdahan Province, northeastern Thailand. It occurs in mixed deciduous and dry evergreen forests, growing on soil covered by a deep layer of leaf litter in shaded to semi-shaded habitats at elevations of 300–400 m (Figure 5).

### 3.9. Preliminary Conservation Assessment

*Typhonium mukdahanense* is currently known only from its type locality in Phu Pha Yol National Park, Mukdahan Province, where it occurs within a very limited area of suitable habitat. Although the study area was carefully surveyed, no additional populations have been documented to date, suggesting that the species may have an extremely restricted distribution. A preliminary assessment following the IUCN Red List Categories and Criteria [18] supports its classification as Critically Endangered (CR) under criterion D, as the known population is estimated to comprise fewer than 50 mature individuals.

## 4. Discussion

*Typhonium mukdahanense* shows the closest morphological affinity to *T. fornicatum*, but is also similar to *T. acetosella* [17]. Both of these species are small, seasonally dormant taxa recorded from northeastern Thailand. *Typhonium mukdahanense* is morphologically most similar to *T. fornicatum*, and the two species share several general features, including a small seasonally dormant habit, clustered leaves, solitary inflorescences, and a slender spadix considerably exceeding the spathe. However, species recognition in this group is better supported by the combination and configuration of several vegetative and reproductive characters than by overall similarity alone. In particular, the new plant population possesses a distinctive combination of leaf morphology, spathe-limb structure, stigma coloration, staminode arrangement, appendix orientation, and fruit morphology that is not present in *T. fornicatum*.

Among these morphological characters, the spathe limb structure and the organization of the sterile interstice provide especially clear distinctions. *Typhonium fornicatum* was originally characterized by its fornicate spathe limb and spreading-declinate appendix, with the fornicate limb regarded as one of its principal distinguishing features. In contrast, the spathe limb of *T. mukdahanense* is triangular and reflexed, with the adaxial surface facing upwards, and its appendix is only slightly curved rather than spreading-declinate. These differences concern the orientation and architecture of the reproductive structure rather than only its dimensions and are therefore useful for distinguishing flowering plants in the natural habitat.

The sterile interstice provides an additional diagnostic distinction. *Typhonium fornicatum* has only three staminodes arranged in a single whorl on the lower portion of the sterile interstice; the original description specifically emphasized this unusually low number of staminodes as a diagnostic feature of that species. Its staminodes are directed upwards. By contrast, *T. mukdahanense* has 10–14 staminodes arranged in two whorls and directed downwards. The difference therefore involves not merely staminode number but also their arrangement and orientation, providing a discrete set of floral characters that can be readily evaluated during anthesis.

Stigma coloration further reinforces this separation. The stigma of *T. mukdahanense* is dark red with a conspicuous cream-colored depressed center, whereas that of *T. fornicatum* is uniformly white. Because this distinction occurs together with marked differences in spathe-limb form and staminode organization, it provides additional support for recognizing the new plant population as morphologically coherent and distinct rather than representing variation within *T. fornicatum*.

Vegetative morphology also facilitates recognition, although these characters are best interpreted together with the reproductive features. *Typhonium mukdahanense* has relatively broad, frequently hastate to trisect leaf blades with 5–8 primary lateral veins per side, whereas *T. fornicatum* has smaller narrowly ovate, ovate, or hastate blades with only 3–4 primary lateral veins per side. Thus, leaf shape and venation provide useful field characters for identifying vegetative individuals, while the floral characters provide stronger confirmation when plants are in flower.

Fruit morphology provides an independent reproductive character supporting the distinction. The berries of *T. mukdahanense* are pyriform and irregularly mottled or speckled with reddish- to purplish-brown markings. In *T. fornicatum*, the berries are clavate, with a subglobose or ovoid head and darker brown mottling. The persistence of morphological differences from the flowering stage through fruiting is particularly useful because it demonstrates that the distinction between the two taxa is expressed across different reproductive stages.The combination of these characters, together with the pyriform mottled berries of *T. mukdahanense*, enables the two species to be distinguished even though they share a generally similar habit.

Taken together, these observations indicate that the taxonomic distinction of *T. mukdahanense* is not based on a single variable feature or on differences in size alone. Rather, it is supported by a consistent combination of characters expressed in the vegetative organs, inflorescence architecture, floral structures, and fruits. Particularly informative are the triangular reflexed spathe limb, dark red stigma with a cream-colored depressed center, 10–14 downward-directed staminodes arranged in two whorls, non-spreading appendix, and pyriform mottled berries. This combination is not found in *T. fornicatum* or *T. acetosella* and therefore provides a practical and morphologically coherent basis for recognizing *T. mukdahanense* as a distinct species.

In comparison with another morphologically similar species, *T. acetosella*, a Thai and Indochinese taxon, *T. mukdahanense* differs by having broader hastate to trisect, dull green leaf blades and longer pistillate zone (2.4–2.8 mm long). In contrast, *T. acetosella* has green, grass-like entire or deeply trilobed leaves with a very narrow linear anterior lobe, shorter pistillate zone (ca. 2 mm long). These stable differences in vegetative, floral, and fruit characters support the recognition of the Mukdahan population as a distinct species. A diagnostic key is presented below to distinguish the new species from other morphologically similar taxa.
1Staminodes three, arranged in a single whorl and directed upwards*T. fornicatum*1Staminodes more than three, arranged in two whorls and directed downwards or slightly downwards22Leaf blades dull green*T. mukdahanense*2Leaf blades green*T. acetosella*

## Figures and Tables

**Table 1:** Comparative morphology of new species to most similar species, *Typhonium fornicatum* P. Saensouk, K.Z. Hein & Saensouk.

Characters	*Typhonium* *mukdahanense*	*Typhonium* *fornicatum*
Plant height	up to 18 cm	up to 10 cm tall
Petioles	7–16 cm long, 1–2 mm in diameter	8.0–9.0 cm long, ca. 0.2 cm in diameter
Leaf blade	8–12 × 6.5–9.5 cm, hastate or trisect or rarely ovate; thinly chartaceous, adaxially dull green, abaxially more dull green	5.0–6.5 × 2.6–2.8 cm, narrowly ovate to ovate, or hastate, thinly coriaceous, adaxially medium green, abaxially pale green
Posterior lobes pointing	outwards	downwards or outwards
Primary lateral vein number	5–8 per side of midrib	3–4 per side of midrib
Peduncle	2–2.2 cm long, 1.5–1.8 mm in diameter, white or pinkish	2.5–4.5 cm long, ca. 0.2 cm in diameter, white
Spathe tube size and shape	12–15 mm long, 7.6–7.9 mm in diameter, symmetrical or slightly asymmetrical ovoid	0.8–0.9 cm long, 0.8–0.9 cm in diameter, obliquely ovoid
Spathe tube color	pinkish orange externally, tinged and streaked purple; internally whitish to pale cream, faintly purplish.	externally pinkish white with a dense dark purple mottling, internally purplish red;
Spathe limb size and shape	12–15 mm long, 7.6–7.9 mm wide at base, linear-triangular, upper part erects and then the spathe limb reflexed, with the adaxial surface facing upward and the abaxial surface facing downward	3.4–2.9 cm long, ca. 0.8 cm wide at base, narrowly lanceolate, upper part fornicate, margins sinuate
Spathe limb color	externally pinkish orange externally, tinged and streaked purple, internally whitish to pale cream, faintly purplish	Externally greenish white with a dense dark purple mottling, internally purplish red
Spadix	9.5–10 cm long	ca. 7.0 cm long
Pistillate zone	2.4–2.8 mm long, 3.8–4.1 mm in diameter at the base, shortly conical, with 4 rows of congested pistils	1.7–2.0 mm long, ca. 2.0 mm in diameter at the base, shortly conical or hemispheroid, with 3 rows of congested pistils
Ovary	0.95–1.1 mm high, 0.6–0.7 mm in diameter, clavate-oblong	ca. 0.6 mm high, ca. 0.6 mm in diameter, obovoid
Stigma	0.5–0.55 mm in diameter, discoid with slightly depressed at center, dark red with cream center	ca. 0.3 mm in diameter, discoid, white
Sterile interstice	10–11.5 mm long, ca. 0.8–0.9 mm in diameter, lower part covered with two whorls of 10−14 staminodes	5–7 mm long, ca. 0.7 mm in diameter, lower part covered with only a single whorl of 3 staminodes
Staminodes	Clavate–falcate, cream or ivory, with a slightly blunt apex and directed downwards	clavate fusiform, yellow, apex obtuse, pointing upwards
Staminate zone	5–6 mm long, 2–2.1 mm in diameter, cylindrical to subcylindrical	ca. 2.5 mm long, ca. 2.7 mm in diameter, shortly subcylindrical
Appendix	8–9 cm long, 0.8–1 mm in diameter, slightly curved, not spreading–declinate, pale cream to very pale creamy brown	5.8–6.0 cm long, 0.8–1.0 mm in diameter at base, spreading–declinate, orange
Berries	pyriform 0.4–0.5 cm long, 0.2–0.3 mm diameter at widest point; color: cream or whitish, or pinkish to pale purplish-brown, irregularly mottled or speckled with reddish-brown to purplish-brown markings	clavate with subglobose or ovoid head, 0.4–0.5 cm long, ca. 0.2 cm at widest point, brownish white with darker brown mottling

## Data Availability

The original contributions presented in this study are included in the article. Further inquiries can be directed to the corresponding author(s).
